# The role of *clockwork orange* in the circadian clock of the cricket *Gryllus bimaculatus*

**DOI:** 10.1186/s40851-020-00166-4

**Published:** 2020-11-11

**Authors:** Yasuaki Tomiyama, Tsugumichi Shinohara, Mirai Matsuka, Tetsuya Bando, Taro Mito, Kenji Tomioka

**Affiliations:** 1 Graduate School of Natural Science and Technology, Okayama University, Okayama, 700-8530 Japan; 2 Graduate School of Medicine, Dentistry and Pharmaceutical Science, Okayama University, Okayama, 700-8558 Japan; 3 Graduate School of Technology, Industrial and Social Sciences, Tokushima University, Tokushima, 770-8513 Japan

**Keywords:** Circadian clock, Clockwork orange, Clock gene, Cricket, *cry2*, Molecular oscillation, Locomotor rhythm

## Abstract

The circadian clock generates rhythms of approximately 24 h through periodic expression of the clock genes. In insects, the major clock genes *period* (*per*) and *timeless* (*tim*) are rhythmically expressed upon their transactivation by CLOCK/CYCLE, with peak levels in the early night. In *Drosophila*, *clockwork orange* (*cwo*) is known to inhibit the transcription of *per* and *tim* during the daytime to enhance the amplitude of the rhythm, but its function in other insects is largely unknown. In this study, we investigated the role of *cwo* in the clock mechanism of the cricket *Gryllus bimaculatus*. The results of quantitative RT-PCR showed that under a light/dark (LD) cycle, *cwo* is rhythmically expressed in the optic lobe (lamina-medulla complex) and peaks during the night. When *cwo* was knocked down via RNA interference (RNAi), some crickets lost their locomotor rhythm, while others maintained a rhythm but exhibited a longer free-running period under constant darkness (DD). In *cwo*^RNAi^ crickets, all clock genes except for *cryptochrome 2* (*cry2*) showed arrhythmic expression under DD; under LD, some of the clock genes showed higher mRNA levels, and *tim* showed rhythmic expression with a delayed phase. Based on these results, we propose that *cwo* plays an important role in the cricket circadian clock.

## Introduction

Most insects exhibit daily rhythms in their physiology, including in their locomotor activity. The rhythms are driven by an endogenous oscillatory mechanism called the circadian clock, which generates approximately 24-h rhythms that persist in the absence of environmental cues [1]. The clock is based on the rhythmic expression of clock genes such as *per*, *tim*, *Clock* (*Clk*), and *cycle* (*cyc*). It is generally thought that the CLOCK (CLK)/CYCLE (CYC) heterodimer activates the transcription of *per* and *tim* by binding to the E-box located upstream of their promoter region [1, 2]. The protein products of *per* and *tim* accumulate during the night, form PER/TIM heterodimers and enter the nucleus to inhibit the transcriptional activity of CLK/CYC late at night. In the fruit fly *Drosophila melanogaster*, the transcriptional activator CLK is also rhythmically expressed by a mechanism that includes *vrille* (*vri*) and *Par domain protein 1ɛ* (*Pdp1ɛ*) [3, 4]. Both *vri* and *Pdp1ɛ* are transactivated by CLK/CYC through the E-box in the late day to early night, similar to *per* and *tim*. Soon after, the transcribed *vri* mRNA is translated to its product protein VRI, which suppresses the transcription of *Clk* during the night, while *Pdp1ɛ* is translated later and activates *Clk* transcription in the late night to early day [3–5]. This mechanism leads to the rhythmic expression of CLK with peak levels during the day.

The *per/tim* oscillatory loop is known to receive fine tuning by *clockwork orange* (*cwo*) in *Drosophila*, which is a clock gene that forms a feedback loop independent of the *per*/*tim* loop [6–8]. *cwo* is rhythmically expressed under the regulation of CLK/CYC with a peak during the night, and its protein product CWO is thought to bind the E-box as a competitor of CLK/CYC, inhibiting the expression of E-box regulated genes, including *per*, *tim*, and *cwo* itself [9]. For *per* and *tim*, CWO function terminates their transcription late at night and suppresses their transcription during the day, causing the production of a higher oscillation amplitude of transcription. A similar function has also been hypothesized for the mammalian homologs, the *Differentiated embryo chondrocyte* (*Dec*) genes [10, 11]. *Drosophila* CWO has also been suggested to activate the transcription of *per*, *tim*, *vri*, and *Pdp1ɛ,* which are target genes of CLK/CYC, and to be involved in posttranslational control of clock proteins [12]. However, the detailed mechanisms for these functions of *cwo* have yet to be explored.

Although *cwo* has been found in some other insects, including the monarch butterfly *Danaus plexippus*, the fire ant *Solenopsis invicta*, and the jewel wasp *Nasonia vitripennis* [13–15], little is known about its function in insect species other than *Drosophila*. To better understand the role of *cwo*, comparative studies using different phylogenetic classes of insects are required, as there are considerable differences in the oscillatory mechanism of the clock among insects [1, 2]. Here, we investigated the role of *cwo* in the cricket *Gryllus bimaculatus*. The oscillatory mechanism of this hemimetabolous insect differs from that of *Drosophila* in several aspects. Instead of *Clk*, *cyc* is rhythmically expressed, while *Clk* is rhythmically expressed when *cyc* is downregulated [16]. In addition to the *per/tim* loop, the mechanism includes *cry* genes that form another feedback loop, which can oscillate independently of the *per*/*tim* loop [17]. In this study, we first detected the presence of the *cwo* gene in *G. bimaculatus* and then examined its role in both behavioral rhythms and molecular oscillatory mechanisms using RNA interference (RNAi). We found that *cwo* plays an essential role in the molecular oscillatory mechanism of *G. bimaculatus* but that there is a compensatory mechanism that can retain behavioral rhythmicity even when the function of *cwo* is disrupted.

## Materials and methods

### Experimental animals

All experiments were performed with adult male crickets (*Gryllus bimaculatus*) that were reared in the laboratory or purchased. The crickets were kept under controlled conditions of 12 h light and 12 h darkness (LD 12:12, light: 0600–1800, Japan Standard Time) at a constant temperature of 25 ± 1.0 °C.

### cDNA cloning

We first searched for *cwo*, *E75*, and *HR3* genes in our RNA-seq data. The sequence data reported for other insect species were used for these searches. The cDNA fragments of the identified genes were obtained via RT-PCR as follows. Total RNA was extracted with TRIzol® Reagent (Ambion, Austin, TX, USA) from 10 adult optic lobes consisting of lamina and medulla neuropiles collected at ZT 6 (ZT stands for zeitgeber time and ZT0 corresponds to lights-on and ZT12 to lights-off). Total RNA (4.5 μg) was used for reverse transcription to obtain cDNA using the PrimeScript® RT reagent Kit (Takara, Otsu, Japan). Using single-stranded cDNA as a template, we performed PCR with EmeraldAmp® PCR Master Mix (Takara) and the primers listed in Table 1. The PCR conditions employed were 40 cycles of 30 s at 95 °C for denaturation, 30 s at 55 °C for annealing, and 1 min 30 s at 72 °C for extension. The amplified sequences were analyzed by BLAST (https://blast.ncbi.nlm.nih.gov/Blast.cgi).

**Table 1:** PCR primers used for quantitative RT-PCR and dsRNA synthesis. The primers tagged with T7 or T3 promoter sequences were used for PCR amplification for dsRNA synthesis. T7 and T3 sequences are underlined

Primers	Forward	Reverse
For qPCR		
*per*	5′-AAGCAAGCAAGCATCCTCAT-3′	5′-CTGAGAAAGGAGGCCACAAG-3′
*tim*	5′-GATTATGAAGTCTGTGATGATTGG-3′	5′-AGCATTGGAGAGAACTGAAGAGGT-3′
*cry2*	5′-AGCACCATCACACACTTCACA-3′	5′-ACACTCAGCGCAATCCACAC-3′
*Clk*	5′-AATGACCGTAGTCGAGAAAGTGAAG-3′	5′-TTGCGATGATTGAGGTTGTTG-3′
*cyc*	5′-GGCCGAAGCTCATAAAGTGG-3′	5′-AACCGCACAAAGGAACCATC-3′
*vri*	5′-TCAGCGTGGAGCAAGTGATG-3′	5′-GGGTACAGCAGCGAGTGTTG-3′
*Pdp1*	5′-TCCCGACGACAAGAAGGAG-3´	5′-AGCGTCTTGTCCCAGAGGTTG-3′
*E75*	5′-CACACGCAAGTGGAGGACA-3´	5′-TTTTGTGCGGCTTGTAGGC-3´
*HR3*	5′-CATGTTGTACCCATCAAAGGTG-3´	5′-TGTGGAGAGCTGGAAACTCC-3´
*rpl18a*	5′-GCTCCGGATTACATCGTTGC-3′	5′-GCCAAATGCCGAAGTTCTTG-3′
For dsRNA synthesis		
*per*	5′-TAATACGACTCACTATAGGGATGTGGCTTGGAAGATCATT-3′	5′-AATTAACCCTCACTAAAGGGTCTCCTTAAGCAAATTCTCA-3′
*tim*	5′-AATTAACCCTCACTAAAGGGGTAAAGAAGATAGAGAGTAT-3′	5′-AATTAACCCTCACTAAAGGGTTGGAGAGAACTGAAGAGGT-3′
*cry2*	5′-TAATACGACTCACTATAGGGAAGCACACTGTGCATTGGTT-3´	5′-AATTAACCCTCACTAAAGGGCCGTTCTTTTCGATGATGCT-3´
*Clk*	5′-TAATACGACTCACTATAGGGTCATAATGAGTTGAGTTCT-3′	5′-TAATACGACTCACTATAGGGAAGGGGTGTCTGTAATCTT-3′
*cyc*	5′-TAATACGACTCACTATAGGGCGTGCACTCGTACACTGAGG-3′	5′-AATTAACCCTCACTAAAGGGAGGTTCTGCTGCTTCTTTCG-3′
*DsRed2*	5′-TAATACGACTCACTATAGGGTCATCACCGAGTTCATGCG-3′	5′-TAATACGACTCACTATAGGGCTACAGGAACAGGTGGTGGC-3′

### RNA measurement

qPCR and RT-PCR were used to measure mRNA levels. Total RNA was extracted and purified from 2 to 6 optic lobes of adult male crickets with TRIzol Reagent (Invitrogen, Carlsbad, CA, USA). To remove genomic DNA contamination, the total RNA was treated with DNase I (Invitrogen). Approximately 500 ng of total RNA from each sample was reverse transcribed using random hexamers and PrimeScript RT reagent Kit (Takara). Real-time PCR was performed with the Mx3000P Real-Time PCR System (Stratagene, La Jolla, CA, USA) using FastStart Universal SYBR Green Master (Roche, Tokyo, Japan), including SYBR Green and primers designed for *cwo*, *per* (GenBank/EMBL/DDBJ Accession No. BAG48878), *tim* (BAJ16356), *Clk* (AB738083), *cyc* (AB762416), *vri* (LC512907), *Pdp1* (LC512908), *E75* (LC536674), *HR3* (LC536673), and *rpl18a* (DC448653) (Table 1). In all cases, a single expected amplicon was confirmed via melting analysis. The quantification was performed based on a standard curve obtained with a known amount of template. The results were analyzed using the software associated with the instrument. The values were then normalized with those of *rpl18a* at each time point. The results of 3–6 independent experiments were used to calculate the mean ± SEM.

### RNAi

Double-stranded RNA (dsRNA) for the cricket clock genes *cwo*, *per*, *tim*, *cry2*, *Clk*, and *cyc* and for the control gene *DsRed2* derived from a coral species (*Discosoma* sp.), were synthesized using a MEGAscript High Yield Transcription kit (Ambion, Austin, TX, USA). For the clock genes, cDNAs prepared as described above were used as templates for PCR, which was performed using ExTaq DNA polymerase (Takara). The T7- or T3-containing primers that were used are listed in Table 1. The amplified fragments of *cwo* (128 bp), *per* (456 bp), *tim* (519 bp), *cry2* (422 bp), *Clk* (407 bp), and *cyc* (450 bp) were extracted with phenol/chloroform, precipitated with ethanol, and then resuspended in Ultra Pure Water (Invitrogen). For *DsRed2* dsRNA, the linearized *DsRed2* fragment was amplified from pDsRed2-N1 (Clontech, Mountain View, CA, USA) using the primers shown in Table 1. Using each of these linearized fragments as a template, RNA was synthesized with T7 or T3 RNA polymerase. Synthesized RNAs were extracted with phenol/chloroform, precipitated with isopropanol, and suspended in 50 μl of TE solution. The yield and quality of the RNA were assessed by measuring the absorbance with a spectrophotometer (GeneQuant Pro, Amersham Bioscience, Piscataway, NJ, USA), and equal amounts of sense and antisense RNA were mixed. The RNAs were denatured for 5 min at 100 °C and were gradually cooled to room temperature for annealing. After ethanol precipitation, the dsRNA obtained was suspended in Ultra Pure Water (Invitrogen) with the final concentration adjusted to 20 μM. The dsRNA solution was stored at − 80 °C until use. The dsRNA solution (760 nl) was injected into the abdomen of adult crickets anesthetized with CO_2_ using a nanoliter injector (WPI, Sarasota, FL, USA).

### Locomotor activity recording

The locomotor activities of the crickets were recorded using the method described previously [18]. In brief, the adult crickets were individually housed in a transparent plastic box (18 × 9 × 4.5 cm) containing a rocking substratum. The movement of crickets resulted in the movement of the substratum, which was recorded every 6 min by a computerized system. Food and water were provided ad libitum. The actographs were placed in an incubator in which light was provided via a cool white fluorescent lamp connected to an electric timer. The raw data were displayed in conventional double-plotted actograms to judge activity patterns and were statistically analyzed using the chi-square periodogram [19] in Actogram J (freely available at http://actogramj.neurofly.de/) [20]. If a peak of the periodogram appeared above the 0.05 confidence level, with the power value (height of the peak above the confidence level) greater than or equal to 10 and the width of the peak greater than or equal to 2, then the period of the peak was designated as statistically significant [21].

### Statistics

The differences in mean mRNA levels between different time points were compared using one-way analysis of variance (ANOVA) followed by a post hoc Tukey’s test. We also used CircWave (ver. 1.4) (available at http://www.rug.nl/fwn/onderzoek/programmas/biologie/chronobiologie/downloads/index) to determine the significance of daily and circadian rhythmicity. When the results of both ANOVA and CircWave analysis were statistically significant, the rhythm was designated as significant. When the result of only one analysis was significant, the pattern was designated quasi-rhythmic. To compare the means of two groups, a t-test was used. The mRNA levels of crickets treated with dsRNA targeting clock genes were compared at each ZT or CT (CT stands for circadian time and CT0 corresponds to projected lights-on and CT12 to projected lights-off) with a control treated with ds*DsRed2* with ANOVA followed by Dunnett’s test. The significance level was set at *P* < 0.05 for all statistics.

## Results

### Molecular cloning and structural analysis of *cwo*

**Fig. 1:**
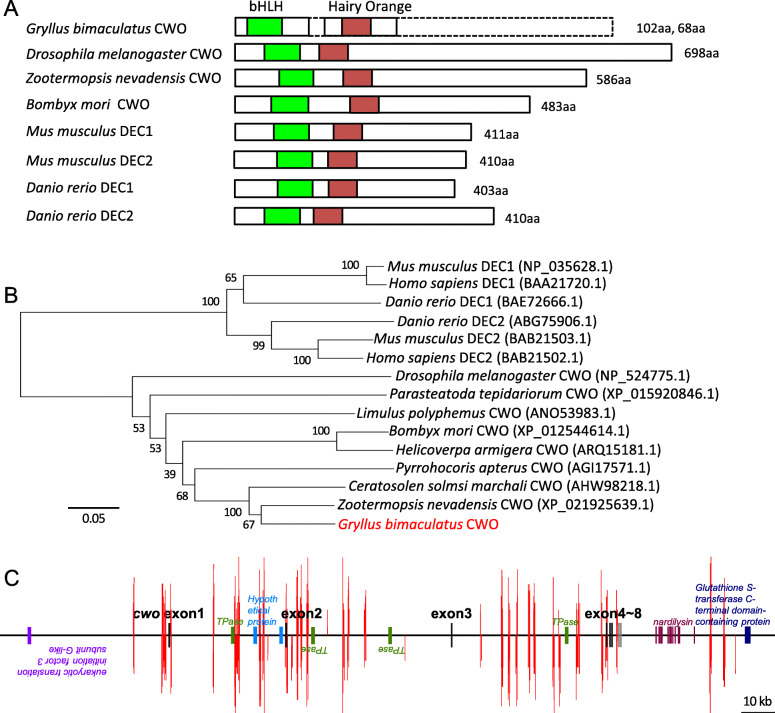
Structural and phylogenetic analysis of *Gryllus bimaculatus clockwork orange* (*Gb’cwo*). **a**: Schematic structure of various CWO or DEC proteins, comparing the organization of the bHLH and Hairy ORANGE domains. **b**: A phylogenetic neighbor-joining tree of known insect CWO proteins and mammalian homologues (DEC proteins). The GenBank accession numbers are indicated in brackets. The reference bar indicates distance as the number of amino acid substitutions per site. **c**: The deduced structure of the *Gb’cwo* gene. Exons 1–8 of *Gb’cwo* are indicated by black bars, and other genes located near *Gb’cwo* are shown in different color bars. Red bars indicate the E-boxes located upstream, downstream, and in intron regions of *Gb’cwo*. See text for details

To obtain a cDNA fragment of *cwo*, we searched for a sequence homologous to known sequences of insect *cwo* genes in our RNA-seq data. We found two fragments, one encoding a 172 aa long protein (GenBank/EMBL/DDBJ Accession No. LC536675), including a bHLH domain, and the other encoding a 123 aa long protein (LC536676), including a Hairy Orange domain (Fig. 1a). We confirmed the sequences by DNA sequencing, followed by RT-PCR, using the primers synthesized for fragment amplification. A BLAST database search indicated that the amino acid sequence of the bHLH domain of *Gryllus bimaculatus* CWO (*Gb’*CWO) has 88.7–96.2% identity and that of the Hairy Orange domain has 34.1–92.5% identity with the amino acid sequences of known insect CWOs, including those of the termite *Zootermopsis nevadensis* (XP_021925639.1), moth *Bombyx mori* (XP_012544614.1), and fruit fly *Drosophila melanogaster* (NP_524775.1) (Table 2). The bHLH domain also has relatively high identity (43.1–46.3%) and similarity (70.4–80.4%) to the vertebrate homologs of CWO, namely, DEC1 and DEC2, in *Danio rerio* (BAE72666.1, ABG75906.1), *Mus musculus* (NP_035628.1, BAB21503.1), and humans (BAA21720.1, BAB21502.1) (Table 2). We thus concluded that the obtained fragments are of *Gryllus bimaculatus cwo* (*Gb’cwo*), which belongs to the bHLH-ORANGE family. A phylogenetic tree based on the amino acid sequences of CWO from known insects and those of DEC1 and DEC2 from some vertebrates revealed that *Gb’*CWO forms a clade with CWOs of other insects and is closely related to that of the termite *Z. nevadensis* (Fig. 1b).

**Table 2:** Identity (%) and similarity (%) of bHLH domain and Hairy ORANGE domain of *Gb’*CWO with other insect CWOs and vertebrate DECs

Species	bHLH domain		Hairy ORANGE domain	
	Identitiy (%)	Similarity (%)	Identity (%)	Similarity (%)
*Zootermopsis nevadensis* CWO	96.2	100	92.5	97.5
*Bombyx mori* CWO	92.5	98.1	57.5	77.5
*Drosophila melanogaster* CWO	88.7	96.2	34.1	48.8
*Danio rerio* DEC1	46.3	70.4	26.2	40.5
*Danio rerio* DEC2	46.3	70.4	26.8	43.9
*Mus musculus* DEC1	43.1	80.4	31.4	68.6
*Mus musculus* DEC2	43.1	80.4	29.7	78.4
*Homo sapiens* DEC1	43.1	80.4	31.4	68.6
*Homo sapiens* DEC2	43.1	80.4	37.8	75.7

We then analyzed the structure of the *Gb’cwo* gene and nearby cis elements. The structure of the *Gb’cwo* gene was deduced using draft genome sequence data of *G. bimaculatus* and the cDNA sequences of known insect *cwo* genes. Figure 1c shows the expected exon/intron structure of the *Gb’cwo* gene. *Gb’cwo* was presumed to consist of 8 exons. We explored cis elements in the 50 kb regions upstream of exon 1 and downstream of exon 8 using Cister (Cis element cluster finder, https://zlab.bu.edu/~mfrith/cister.shtml) and found many E-boxes in both sense and antisense strands, especially within the 10 kb region upstream of exon 1.

### Tissues expressing *cwo*

**Fig. 2:**
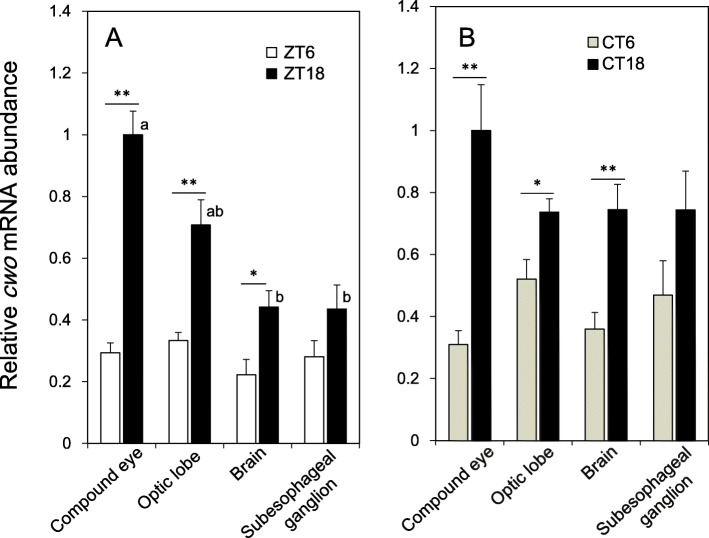
*cwo* expression in the compound eye, optic lobe, brain, and subesophageal ganglion of the cricket *Gryllus bimaculatus* under LD (**a**) and DD (**b**). The mRNA expression levels showed a significant day-night change in the compound eye, optic lobe, and brain, with the levels being significantly higher at night (ZT18) or subjective night (CT18) (* *P* < 0.05, ** *P* < 0.01, t-test). The mRNA level and amplitude were greatest in the compound eye under both LD and DD conditions. The values are shown as relative to the mRNA levels of *rpl18a* and are normalized to the highest value measured in the compound eye. Different letters indicate a significant difference detected by Tukey’s test (*P* < 0.05). See text for details

To determine which tissues express *cwo*, we measured *cwo* mRNA levels in the optic lobe, protocerebral lobe (brain), subesophageal ganglion, and compound eyes by qPCR. The samples were collected at midday (ZT6), midnight (ZT18), subjective midday (CT6), and subjective midnight (CT18). As shown in Fig. 2, *cwo* mRNA was detected in all of these tissues. Under the LD cycle, the expression was highest in the compound eye and lowest in the brain and subesophageal ganglion. The *cwo* RNA levels in the compound eye, optic lobe, and brain changed daily, with the highest amplitude (3.4-fold) in the compound eye (Fig. 2a). The day-night changes in the optic lobe and brain were 1.8-fold and 2.0-fold, respectively. The subesophageal ganglion also showed higher *cwo* levels at night, but the difference was not significant.

Under constant darkness (DD), daily expression profiles were basically reproduced in tissues collected at CT6 and CT18 (Fig. 2b). In the compound eye, optic lobe, and brain, the *cwo* levels were higher at CT18, and the circadian changes were 3.2-fold, 1.4-fold, and 2.1-fold, respectively. The expression in the subesophageal ganglion did not show a significant rhythm.

### Daily expression of *cwo* mRNA

**Fig. 3:**
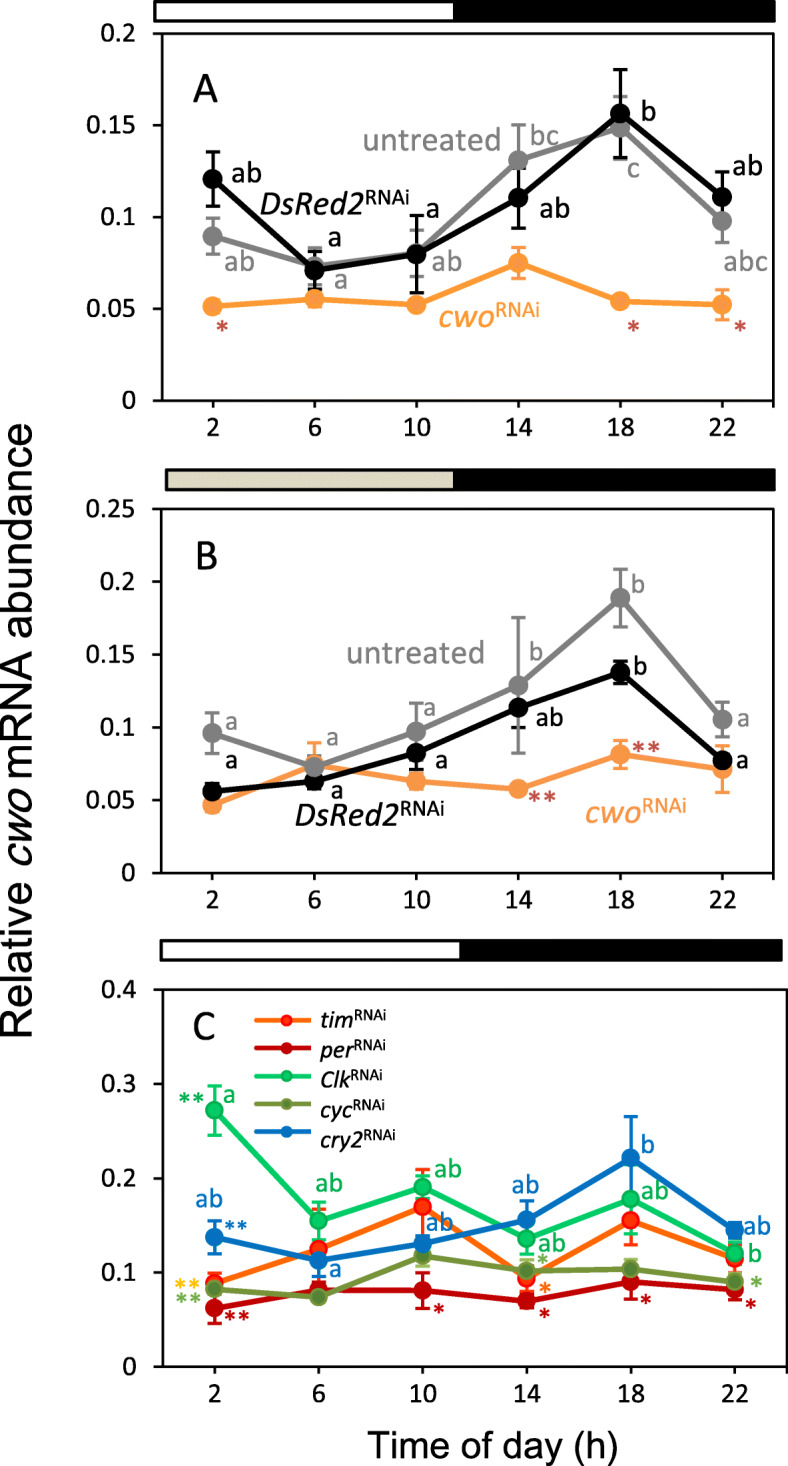
Expression rhythms of *cwo* in the optic lobe of the cricket *Gryllus bimaculatus* and the effects of clock gene RNAi on the expression of *cwo*. **a**: *cwo* is rhythmically expressed in the optic lobe with a peak level at midnight in both untreated (gray) and *DsRed2*^RNAi^-treated crickets (black) under LD. ds*cwo* strongly suppressed *cwo* mRNA levels and eliminated its rhythmic expression (orange). **b**: *cwo* is also rhythmically expressed under DD in the optic lobe of untreated (gray) and *DsRed2*^RNAi^-treated crickets (black). ds*cwo* again suppressed *cwo* mRNA levels and eliminated its rhythmic expression (orange). **c**: Effects of dsRNA of clock genes, *per*, *tim*, *Clk*, *cyc*, and *cry2* on *cwo* expression under LD. *per*^RNAi^*, tim*^RNAi^, and *cyc*^RNAi^ eliminated the daily rhythmic expression of *cwo,* and the suppression was stronger in *per*^RNAi-^ and *cyc*^RNAi^-treated crickets. *Clk*^RNAi^ and *cry2*^RNAi^ did not eliminate the rhythm. *cry2*^RNAi^ treatment had no significant effects on the expression rhythm of *cwo*, while *Clk*^RNAi^ induced its phase shift to peak at ZT2. Asterisks indicate significant differences compared to the control treated with ds*DsRed2* (* *P* < 0.05, ** *P* < 0.01, Dunnett’s test). Different lowercase letters indicate that the values differ significantly from each other (Tukey’s test, *P* < 0.05). White, gray, and black bars above the panel indicate light (white), subjective day (gray), and dark/subjective night (black) fractions, respectively. See text for details

We first examined the expression profile of *cwo* mRNA in the cricket clock tissue, the optic lobe, under LD 12:12. The qPCR results showed that *cwo* mRNA was rhythmically expressed (Fig. 3a, Table 3). It was expressed at a low level during the daytime, with the expression gradually increasing around light-off and peaking in the middle of the night. The profile was similar to those of *per* and *tim* [18, 22] (see also Figs. 5 and 6). A similar expression pattern was observed 2 days after the crickets were transferred to DD (Fig. 3b, Table 3). The mRNA levels were similar between LD and DD conditions.

**Table 3:** Results of statistical analyses of *cwo* expression in untreated crickets and those treated with RNAi of *DsRed2* or clock genes under light-dark cycle (LD) or constant darkness (DD)

Treatment	ANOVA			CircWave
	d.f.	F	P	P
LD				
untreated	5, 60	4.0806	0.0011	0.000185
*DsRed2*^RNAi^	5, 45	3.0001	0.0202	0.007221
*cwo*^RNAi^	5, 17	2.6963	0.057	> 0.05
DD				
untreated	5, 14	5.9411	0.0038	0.001358
*DsRed2*^RNAi^	5, 26	10.7877	0.00001	0.0000
*cwo*^RNAi^	5, 23	1.7097	0.1724	> 0.05
LD				
*tim*^RNAi^	5, 18	1.3930	0.2736	> 0.05
*per*^RNAi^	5, 12	0.5117	0.7626	> 0.05
*Clk*^RNAi^	5, 18	5.8265	0.0023	> 0.05
*cyc*^RNAi^	5, 17	1.5834	0.2179	> 0.05
*cry2*^RNAi^	5, 27	3.0132	0.0274	0.010773

We then examined the effects of RNAi of other clock genes, including *per*, *tim*, *cry2*, *Clk*, and *cyc*, on the mRNA levels of *cwo*. As a control, we tested the effects of RNAi of *DsRed2*. The *cwo* expression profiles in the *DsRed2*^RNAi^-treated crickets were similar to those of the untreated crickets under both LD and DD (Fig. 3a, b), and no significant difference was observed at all ZTs and most CTs (t-test, *P* > 0.05); the exceptions were CT2 and CT22, in which the values were lower than those of untreated crickets (t-test, *P* < 0.05). RNAi of *per*, *tim*, and *cyc* resulted in the loss of daily rhythm of *cwo* expression, and *per*^RNAi^ and *cyc*^RNAi^ downregulated the mRNA levels of *cwo* compared to *DsRed2*^RNAi^, while *Clk*^RNAi^ resulted in a quasi-rhythmic expression of *cwo* with a peak in the early day (Fig. 3c, Table 3). The results suggest that *cwo* is under the regulation of the circadian clock. Interestingly, however, ds*cry2* treatment showed almost no effect on the rhythmic expression of *cwo* (Fig. 3c, Table 3).

### Effects of *cwo* dsRNA treatment on locomotor rhythm

To investigate the role of *cwo* in the cricket clock, we first examined the effects of systemic *cwo*^RNAi^ on the mRNA levels of *cwo* in the optic lobe under LD and DD conditions. Under both conditions, *cwo*^RNAi^ treatment significantly reduced the *cwo* mRNA levels to below or near the basal level in controls treated with *DsRed2*^RNAi^ and eliminated the rhythmic expression of *cwo* that was evident in control crickets (Fig. 3a, b, Table 3).

**Fig. 4:**
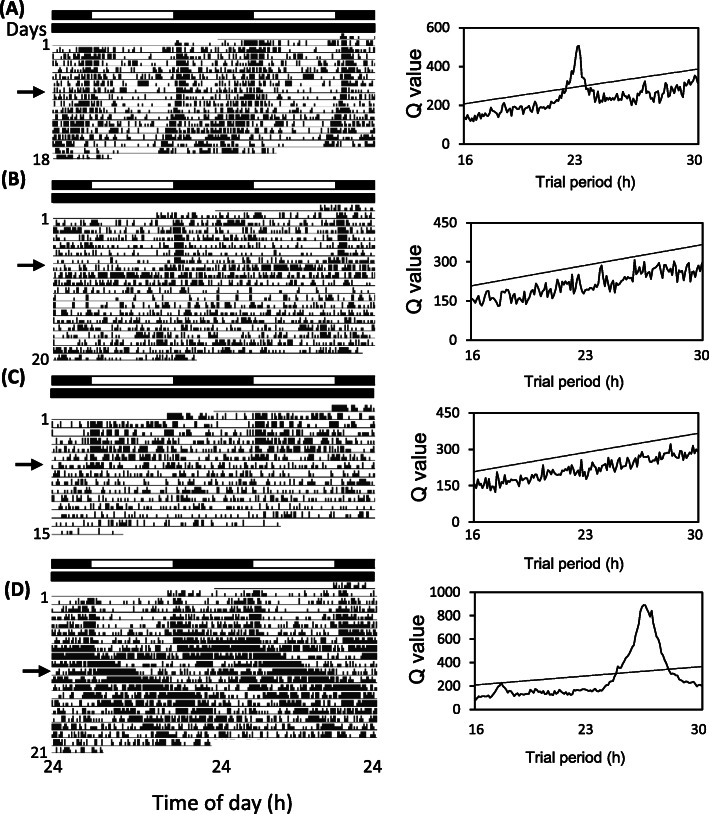
Effects of ds*cwo* on the locomotor rhythm in the cricket *Gryllus bimaculatus* under a light-dark cycle (LD) and in subsequent constant darkness (DD). The left and right panels show the double-plotted actograms and results of chi-square periodogram analysis, respectively. **a**: A representative control cricket treated with ds*DsRed2*, showing a clear nocturnal rhythm in LD and a free-running period slightly shorter than 24 h in the ensuing DD. **b**-**d**: Locomotor rhythms of crickets injected with ds*cwo* in the abdomen (**b**) or compound eyes (**c**, **d**). Most treated crickets showed a nocturnal rhythm (**b**, **d**), but some showed diurnal rhythms (**c**), in LD. Some of the crickets became arrhythmic on transfer to DD (**b**, **c**), while the remaining crickets showed a free-running rhythm with a period longer than 24 h (**d**). Arrows indicate the day of transfer from LD to DD. White and black bars above the actograms indicate light (white) and dark (black) fractions, respectively. See text for details

We then tested the effects of *cwo*^RNAi^ on circadian locomotor rhythms. We injected *cwo* dsRNA into the abdomen or bilateral compound eyes in 22 and 10 adult male crickets, respectively, and recorded their locomotor activity, first under LD conditions for a week and then under DD. Because the results of these two treatments were similar, we pooled the results. We also recorded the locomotor activity of *DsRed2*^RNAi^-treated crickets as a control (*n* = 16). As shown in Fig. 4a, most of the control crickets (*n* = 15) showed a clear nocturnal rhythm in LD and a free-running rhythm with a period that was slightly shorter than 24 h in the ensuing DD, while one cricket became arrhythmic. The average free-running period of the rhythmic crickets was 23.5 ± 0.3 (SD) h. In *cwo*^RNAi^ crickets (*n* = 32), 23 showed a nocturnal rhythm similar to that of controls (Fig. 4b, d), while 9 showed diurnal activity in LD (Fig. 4c). In DD, the 9 diurnal crickets and one nocturnal cricket became arrhythmic, with activity dispersed over 24 h (Fig. 4b, c). The remaining 22 crickets showed a free-running rhythm with a period (24.4 ± 1.5 h) that was significantly longer than that of *DsRed2*^RNAi^ controls (Fig. 4d) (t-test, *P* < 0.05). The proportion of arrhythmic crickets in *cwo*^RNAi^ treatments was significantly higher than that in the *DsRed2*^RNAi^ control (chi-square test, *P* < 0.05).

### Effects of *cwo* dsRNA treatment on the clock molecular machinery

**Fig. 5:**
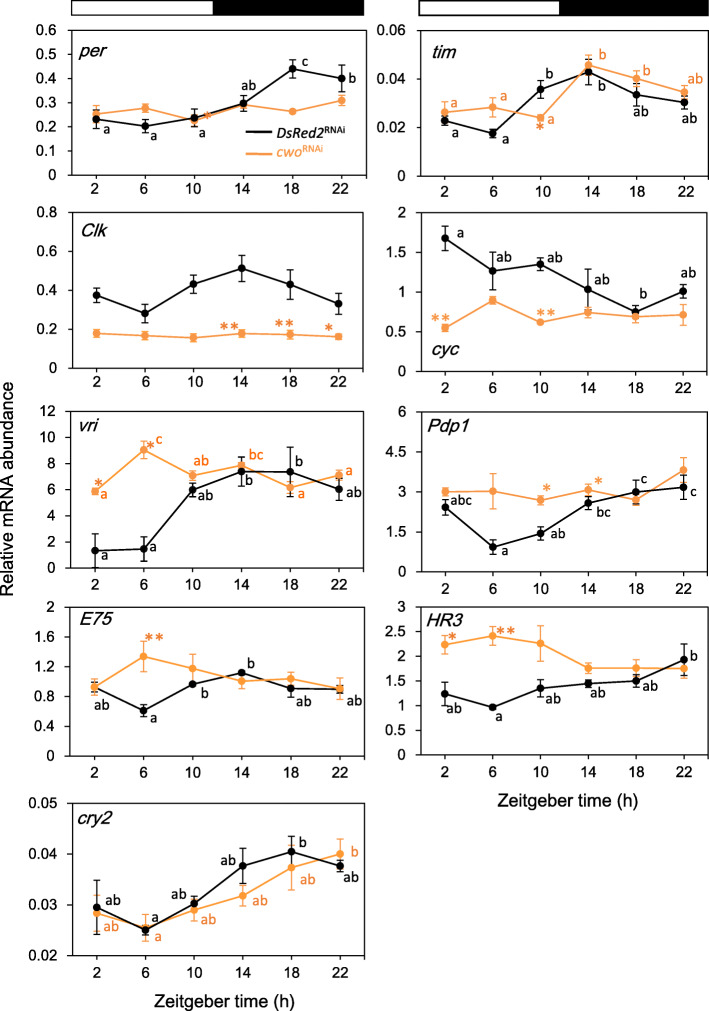
Effects of *cwo* dsRNA on the mRNA expression of clock genes *per*, *tim*, *Clk*, *cyc*, *vri*, *Pdp1*, *E75*, and *HR3* under LD. Orange: *cwo*^RNAi^-treated crickets. Black: *DsRed2*^RNAi^-treated control crickets. Ds*cwo* significantly downregulated *per*, *Clk*, and *cyc* and eliminated the daily rhythmic expression of *per* and *cyc*. Ds*cwo* neither downregulated *tim* and *cry2* nor eliminated their rhythmic expression but induced a slight phase delay. *Vri*, *Pdp1*, *E75*, and *HR3* were significantly upregulated, and their expression stayed at a level near their peak level in *DsRed2*^RNAi^-treated controls or higher. Different letters indicate a significant difference detected by Tukey’s test (*P* < 0.05). Asterisks indicate significant differences compared to the control treated with ds*DsRed2* (* *P* < 0.05, ** *P* < 0.01, t-test). White and black bars above the panel indicate light (white) and dark (black) fractions, respectively. See text for details

**Fig. 6:**
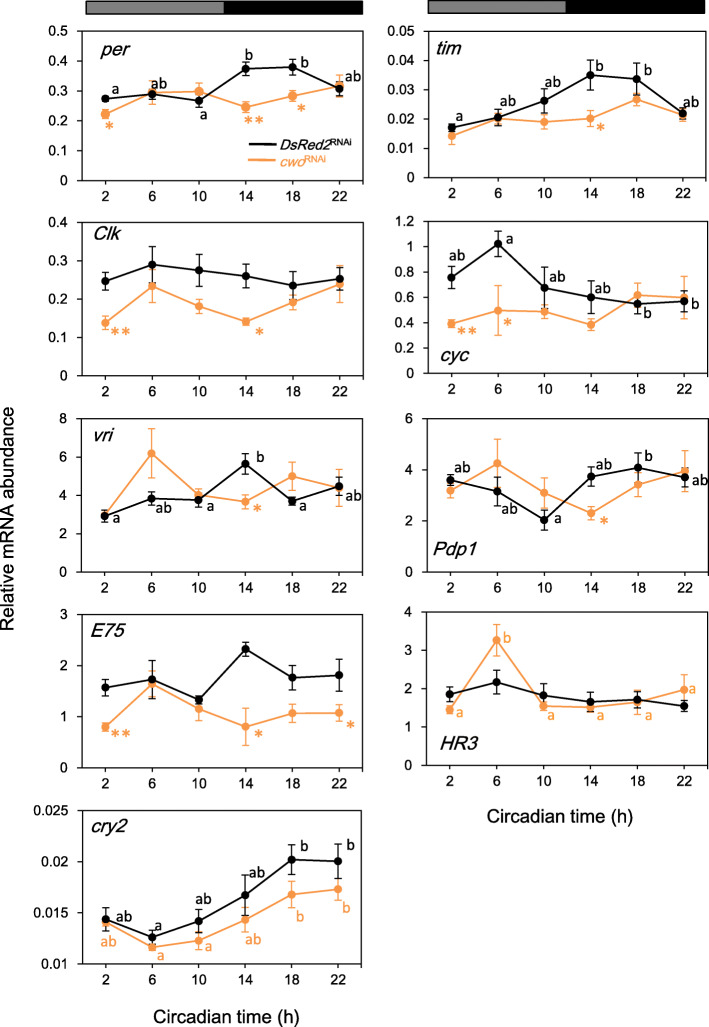
Effects of *cwo* dsRNA on the mRNA expression of clock genes *per*, *tim*, *Clk*, *cyc*, *vri*, *Pdp1*, *E75*, and *HR3* under DD. Orange: *cwo*^RNAi^-treated crickets. Black: *DsRed2*^RNAi^-treated control crickets. Ds*cwo* significantly downregulated *per*, *tim, Clk*, *cyc*, and *E75* and eliminated the daily rhythmic expression of *per*, *tim*, *cyc*, *vri*, and *Pdp1*. *cry2* maintained its rhythmic expression. Different letters indicate a significant difference detected by Tukey’s test (*P* < 0.05). Asterisks indicate significant differences compared to the control treated with ds*DsRed2* (* *P* < 0.05, ** *P* < 0.01, t-test). Gray and black bars above the panel indicate subjective day (white) and subjective night (black), respectively. See text for details

To investigate the role of *cwo* in the clock oscillatory machinery, we examined the effects of *cwo*^RNAi^ on the expression profile of the clock genes *per*, *tim*, *cry2*, *Clk*, *cyc*, *vri*, *Pdp1*, *E75*, and *HR3*. We measured the mRNA levels of the genes in the optic lobe of adult male crickets, which were injected with ds*cwo* in the abdomen and kept under LD or DD. The results are shown in Figs. 5 and 6 and Table 4. Under LD, the mRNA levels of *per*, *tim*, *cyc*, and *cry2* in control crickets treated with *DsRed2*^RNAi^ showed the expression profiles that were previously reported for untreated crickets: *per*, *tim* and *cry2* were rhythmically expressed with a peak during the night, while the levels of *cyc* peaked during the day (Fig. 5, Table 4) [16, 18, 22]. The expression of *Clk* was quasi-rhythmic (Fig. 5, Table 4), although it was previously reported to be constitutively expressed [23]. *vri*, *Pdp1*, and *HR3* were also rhythmically expressed, while *E75* was expressed quasi-rhythmically with a peak during the night (Fig. 5, Table 4). *cwo*^RNAi^ treatment significantly downregulated the expression of *per*, *Clk*, and *cyc* to eliminate their daily rhythms (Fig. 5, Table 4). However, *tim* and *cry2* maintained a clear rhythm of expression, but the rising phase was slightly delayed in *tim,* and the peak was slightly delayed in *cry2* (Fig. 5, Table 4). *vri*, *Pdp1*, *E75*, and *HR3* were upregulated upon *cwo*^RNAi^ treatment. Their transcript levels stayed at levels similar to or higher than their peak levels in *DsRed2*^RNAi^-treated control crickets (Fig. 5).

**Table 4:** Results of statistical analyses of daily clock gene expression in crickets treated with *DsRed2*^RNAi^ (control) or *cwo*^RNAi^ under light-dark cycle (LD) or constant darkness (DD)

Clock genes	Treatment	ANOVA			CircWave
		d.f.	F	P	P
LD					
*per*	*DsRed2*^RNAi^	5, 44	6.4931	0.0001	0.00001
	*cwo*^RNAi^	5, 18	1.8144	0.1607	> 0.05
*tim*	*DsRed2*^RNAi^	5, 16	5.8537	0.0029	0.000896
	*cwo*^RNAi^	5, 17	6.2874	0.0018	0.001774
*Clk*	*DsRed2*^RNAi^	5, 15	2.298	0.0972	0.047724
	*cwo*^RNAi^	5, 17	0.2012	0.9575	> 0.05
*cyc*	*DsRed2*^RNAi^	5, 15	4.0251	0.0162	0.008703
	*cwo*^RNAi^	5, 17	2.6741	0.0584	> 0.05
*vri*	*DsRed2*^RNAi^	5, 16	5.312	0.0046	0.00026
	*cwo*^RNAi^	5, 18	7.5919	0.0005	> 0.05
*Pdp1*	*DsRed2*^RNAi^	5, 48	6.9259	0.0001	0.000004
	*cwo*^RNAi^	5, 18	1.2890	0.3119	> 0.05
*E75*	*DsRed2*^RNAi^	5, 16	4.2222	0.0122	> 0.05
	*cwo*^RNAi^	5, 17	1.3585	0.2883	> 0.05
*HR3*	*DsRed2*^RNAi^	5, 18	2.8840	0.0439	0.031795
	*cwo*^RNAi^	5, 17	2.4046	0.0801	0.007331
*cry2*	*DsRed2*^RNAi^	5, 15	4.2396	0.0133	0.000402
	*cwo*^RNAi^	5, 17	3.2741	0.0298	0.00397
DD					
*per*	*DsRed2*^RNAi^	5, 20	5.6718	0.0020	0.000773
	*cwo*^RNAi^	5, 16	1.6874	0.1947	> 0.05
*tim*	*DsRed2*^RNAi^	5, 26	3.8506	0.0096	0.000482
	*cwo*^RNAi^	5, 14	2.4257	0.0878	> 0.05
*Clk*	*DsRed2*^RNAi^	5, 27	0.3144	0.8999	> 0.05
	*cwo*^RNAi^	5, 17	2.3294	0.0885	> 0.05
*cyc*	*DsRed2*^RNAi^	5, 28	2.9222	0.0303	0.007461
	*cwo*^RNAi^	5, 16	1.0490	0.4235	> 0.05
*vri*	*DsRed2*^RNAi^	5, 25	5.3741	0.0017	0.023523
	*cwo*^RNAi^	5, 13	2.1364	0.1254	> 0.05
*Pdp1*	*DsRed2*^RNAi^	5, 23	2.9852	0.0321	0.020129
	*cwo*^RNAi^	5, 17	1.2542	0.3280	> 0.05
*E75*	*DsRed2*^RNAi^	5, 24	2.2516	0.0818	> 0.05
	*cwo*^RNAi^	5, 18	1.6580	0.1957	> 0.05
*HR3*	*DsRed2*^RNAi^	5, 27	0.7698	0.5798	> 0.05
	*cwo*^RNAi^	5, 17	5.7916	0.0027	> 0.05
*cry2*	*DsRed2*^RNAi^	5, 30	5.1756	0.0015	0
	*cwo*^RNAi^	5, 17	5.3303	0.0040	0.000107

Under DD, the mRNA expression profiles of the clock genes in the *DsRed2*^RNAi^ control crickets were basically similar to those observed under LD, except for *Pdp1* and *HR3*; the former showed a rhythmic expression that peaked at mid-subjective night, while the latter was expressed essentially constitutively (Fig. 6, Table 4). The effects of *cwo*^RNAi^ were similar to those obtained under LD; however, *tim*, *E75*, and *HR3* showed features different from those observed under LD (Fig. 6). Specifically, *tim* lost its oscillation, while *E75* was significantly downregulated, and the levels of *HR3* were similar to those observed in *DsRed2*^RNAi^-treated controls, implying that light plays a certain role in the clock oscillatory mechanism. A fluctuation was observed in the mRNA levels of *vri* and *Pdp1,* but the periodicity of the changes was not significant (Fig. 6, Table 4). Interestingly, *cry2* retained a weak but significant oscillatory expression with a peak in late subjective night, similar to that under LD (Fig. 6, Table 4), suggesting its role in the generation of locomotor rhythm in *cwo*^RNAi^ crickets.

## Discussion

### The *cwo* gene

In the present study, we obtained the partial sequence of *Gb’cwo* from the RNA-seq data and verified its existence by cDNA cloning. Analysis of the sequence revealed that *cwo* of crickets is a member of the bHLH-ORANGE family. Expression analysis of *cwo* with qRT-PCR revealed that it is rhythmically expressed in the compound eye, optic lobe, and brain, suggesting that it is involved in rhythm generation in these tissues (Fig. 2). This result is consistent with our previous findings that the optic lobe is the locus of the circadian clock that controls locomotor rhythms [24], that the compound eye shows circadian rhythms in its sensitivity to light [25], and that the brain shows rhythmic expression of *per* and *tim* [26]. It is also expressed at some level in the subesophageal ganglion, but rather constitutively, suggesting that *cwo* may play a role other than its role in the circadian clock, similar to *per* and *tim* in the *Drosophila* gonads [27], and *vri* in larval molting and metamorphosis in the moth *Helicoverpa armigera* [28]. Further studies may reveal additional, non-clock functions of *cwo*.

### Regulation of *cwo* expression

Our results showed that *cwo* is rhythmically expressed in the optic lobe, the clock tissue of crickets, with a peak in the middle of the night (Fig. 3). This result is consistent with reports from *Drosophila* [6–8, 12] suggesting that *cwo* is under the control of the circadian clock. A similar expression profile has been reported in the monarch butterfly *D. plexippus* [14], while no significant daily *cwo* rhythm has been detected in the wasp *N. vitripennis* [15]. *cyc*^RNAi^ treatment led to strong downregulation of the expression of *cwo* to the basal level of the *DsRed2*^RNAi^ control (Fig. 3c). Considering that *cwo* is transactivated by CLK/CYC through the E-box in *Drosophila* and that there are many E-box elements in the UTR regions of *Gb’cwo* (Fig. 1c), this result suggests that cricket *cwo* is transactivated by a similar mechanism, although we could not exclude the possibility that CYC affects *cwo* expression via non-E-box mediated mechanisms. *Clk*^RNAi^ treatment did not eliminate the *cwo* rhythm but shifted it by 8 h, such that it peaked in the early morning without any reduction in transcript levels (Fig. 3c), whereas in *Clk-*knockout monarch butterflies, *cwo* was expressed at constitutively low levels [14]. This may be explained by the gradual accumulation of *Clk* mRNA that survived RNAi treatment, with the resultant CLK/CYC complexes stimulating *cwo* transcription in a delayed time course, or by transactivation of *cwo* by CYC alone in a delayed manner. Treatment with *Clk*^RNAi^ leads to arrhythmic locomotor activity and terminates the oscillation of the transcript levels of *per* and *tim* [23]. Therefore, as *cwo* oscillation survived the *Clk*^RNAi^ treatment, it is possible that *cwo* has no significant role in rhythm generation. These possibilities should be examined in future studies.

**Fig. 7:**
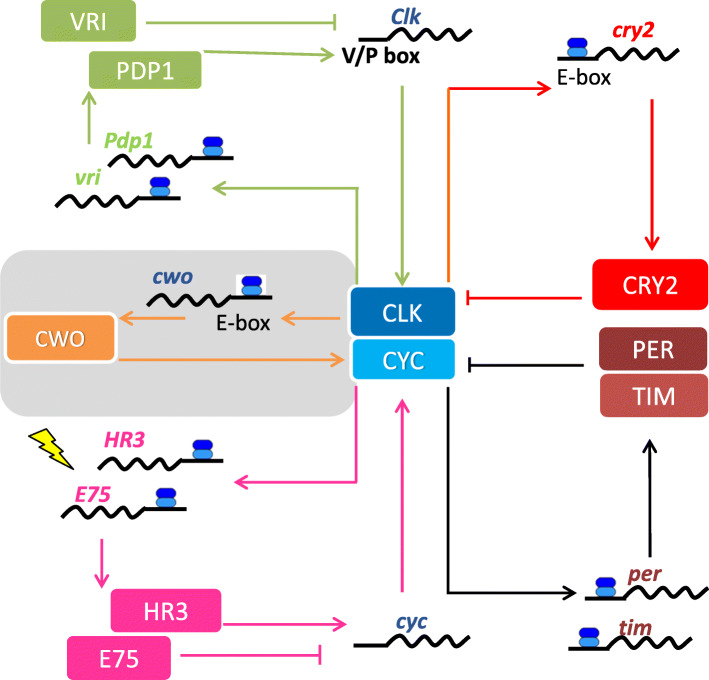
A model of the clock oscillatory mechanism in the cricket *Gryllus bimaculatus*. The clock most likely oscillates based on transcriptional/translational feedback loops. Transcription of *per*, *tim*, *cry2*, *vri*, *Pdp1*, *E75*, and *HR3* is activated by CLK and CYC through the E-box. The protein products PER and TIM form heterodimers and suppress the transcriptional activity of CLK/CYC, while CRY2 also suppresses CLK/CYC through a pathway that is different from that of PER/TIM. Previous results have suggested that both *Clk* and *cyc* could be rhythmically expressed. According to the circadian oscillatory mechanism of *Drosophila* and firebrats [4, 33], VRI and E75 suppress, while PDP1 and HR3 activate, the transcription of *Clk* and *cyc*, respectively. CWO probably enhances the transcription of CLK/CYC target genes through upregulation of *Clk* and *cyc*. When *cwo* is downregulated, light promotes the expression of *HR3* and *E75*. See text for details

RNAi of *per* or *tim* was found to eliminate the daily rhythmic expression of *cwo* (Fig. 3c). This effect may be caused indirectly through complex clock machinery. Since their RNAi downregulates the expression of *Clk* and *cyc* [16, 23], the decrease in the levels of CLK and CYC may in turn result in downregulation of the expression of *cwo*. *per*^RNAi^ induces arrhythmicity in locomotor activity, while *tim*^RNAi^ shortens the free-running period of locomotor rhythms [18, 22]. The maintenance of locomotor rhythm in *tim*^RNAi^ crickets is most likely attributable to oscillation of *cry2* [17]. Thus, *cwo* oscillation may not be required for the *cry2* oscillation. This hypothesis is also supported by the results of this study, which show that *cry2* maintained its rhythmic expression in *cwo*^RNAi^ crickets under both LD and DD (Figs. 5 and 6). The present study also revealed that *cry2*^RNAi^ treatment had almost no effect on the rhythmic expression of *cwo* (Fig. 3c), suggesting that *cry2* oscillation is independent of the main *per*/*tim* loop, including *cwo* (Fig. 7).

### Role of *cwo* in the clock machinery

The role of *cwo* has been extensively studied in *Drosophila* [6–8]. It is a transcription factor belonging to the bHLH-ORANGE family. The lack of *cwo* results in a longer free-running period of locomotor rhythm and a reduced amplitude of *per* and *tim* cycling [12]. It has been shown that CWO binds to the E-box during late night to midday in competition with CLK/CYC to inhibit the transcription of E-box-dependent genes, such as *per* and *tim*, increasing the amplitude of their daily expression rhythms [9].

The results of this study showed that the locomotor rhythm phenotypes of *cwo*^RNAi^ crickets are quite similar to those reported for *cwo*-deficient *Drosophila* mutant flies. They showed either arrhythmic activity or rhythms with longer free-running periods (Fig. 4), suggesting that *cwo* plays an important role in the cricket clock mechanism. The effects of *cwo*^RNAi^ at the molecular level were more severe than those found in *cwo-*deficient flies: under DD, *cwo*^RNAi^ downregulated most E-box-regulated clock genes, including *per*, *tim*, and *E75*, and eliminated the expression rhythm in *per*, *tim, vri, Pdp1*, and *E75* (Fig. 6). These effects may be explained by the regulation of gene transcription by CWO through the E-box-dependent transcription factors CLK and CYC, generating robust rhythmic expression, as in *Drosophila* [12] (Fig. 7). In fact, *cwo*^RNAi^ significantly downregulated the expression of *Clk* and *cyc* and eliminated the rhythmic expression of *cyc* (Fig. 6). Therefore, *cwo* may function as a transcriptional activator (Fig. 7), as has been suggested in *Drosophila* [6–8, 12], although the mechanism of transcriptional activation by CWO is currently unknown. CWO may activate transcription of the E-box mediated clock genes by enhancing the transcriptional activity of CLK and CYC (Fig. 7). Alternatively, it may activate the transcription of *Clk* and *cyc*, which in turn activate the E-box mediated clock genes.

Our data were obtained via RNAi-mediated gene silencing experiments and were not fully compatible with the results obtained by genetic manipulation in *Drosophila*. Nonetheless, the regulatory role of CWO in each of the clock-relevant genes may be different between crickets and *Drosophila*. Further studies are required to resolve this issue.

Importantly, *cry2* maintained its rhythmic expression upon *cwo*^RNAi^ treatment, even after the rhythmic expression of all other clock genes was interrupted (Figs. 5 and 6). The maintenance of locomotor rhythms in *cwo*^RNAi^ crickets is most likely attributable to the *cry2* rhythm. This finding is consistent with our previously proposed hypothesis that *cry2* forms a transcriptional/translational feedback loop that can function independently of the *per/tim* oscillatory loop in the cricket [17] (Fig. 7).

Interestingly, *cwo*^RNAi^ treatment revealed that light modulates the oscillatory system. The treatment reduced the transcript levels of *E75* and had no significant effect on the transcript level of *HR3* under DD, while those levels were significantly higher than the control under LD (Figs. 5 and 6). These observations suggest that light somehow modulates the transcription of these genes. In addition, *tim* was rhythmically expressed under LD at a level similar to that in control crickets treated with *DsRed2*^RNAi^ but with the rising phase slightly delayed (Fig. 5). *tim* is known to maintain its rhythmic expression even when other clock genes are arrhythmically expressed at low levels due to double RNAi of *cry1* and *cry2* [17]. Thus, the mechanism regulating *tim* cycling may differ from those for other E-box mediated clock genes. In *cwo*-deficient *Drosophila* mutants, the falling phase of *tim* is reported to be slightly delayed [6], suggesting that the mechanism of *tim* regulation by *cwo* differs between the two species. Although the mechanisms underlying these light-dependent changes in clock gene expression are currently unclear, they contribute to the maintenance of a molecular rhythm in *cwo*^RNAi^ crickets to generate robust daily behavioral rhythms under LD, together with the *cry2* loop, which persists under both LD and DD. While further study is necessary to clarify the underlying mechanism, our results are reminiscent of the light-dependent induction of *tim* expression in *Drosophila* [29].

Apart from its function in the oscillatory mechanism, the mammalian *cwo* homolog *Dec1* is known to play an essential role in the phase resetting mechanism of peripheral clocks [30]. In this mechanism, *Dec1* is induced through TGF/ALK5/SMAD signaling but independent of *Per1*/*Per2* induction, which is essential for light-evoked phase resetting [31]. Although we currently have no direct evidence for the involvement of *cwo* in clock phase resetting in cricket, this issue should be addressed in future studies since the cricket clock is known to be reset by a nonphotic but rather temperature-dependent mechanism [32].

## Conclusions

In this study, we have shown that *cwo* in the cricket *Gryllus bimaculatus* is a clock gene belonging to the bHLH-ORANGE family and is rhythmically expressed in the clock tissue, the optic lobes, and peaks during the night under the LD cycle. *Cwo* plays an important role in the regulation of behavioral rhythms, as *cwo*^RNAi^ resulted in arrhythmicity or elongation of the free-running period of locomotor rhythms. This alteration in behavioral rhythms is most likely caused by changes in the molecular oscillatory mechanism; in *cwo*^RNAi^ crickets, the expression of most clock genes became arrhythmic, and *cry2* alone retained rhythmic expression under DD. Based on these results, we propose that *cwo* is a component of the *per/tim* oscillatory loop (Fig. 7). To our knowledge, this is the first study on the function of *cwo* in insects other than *Drosophila*. There are some differences in the role of *cwo* between *Drosophila* and crickets. Since crickets are hemimetabolous and are phylogenetically more basal than *Drosophila*, the role of cricket *cwo* may be more ancestral than that in *Drosophila*. To understand the general role of *cwo* in insect clocks, its functions should be compared among different groups of insects.

## Data Availability

The datasets supporting the conclusions of this article are included within the article.
